# Attention-Deficit/Hyperactivity Disorder (ADHD) In Childhood, A Warning Sign Of Bipolar Disorder?

**DOI:** 10.1192/j.eurpsy.2025.663

**Published:** 2025-08-26

**Authors:** R. Tarazhi, V. Alikaj, V. Skendi

**Affiliations:** 1 Child and Adolescent Psychiatry, Psychiatric Hospital, Tirana, Albania

## Abstract

**Introduction:**

Early-onset bipolar disorder (BD) and attention-deficit hyperactivity disorder (ADHD) have recently been the subject of a highly controversial debate, due to theories regarding the underlying pathophysiological processes and a clinical overlap of symptoms. Epidemiological data, clinical aspect, neuroimaging, neurochemical and genetic studies suggest that there may be a possible relationship between biological factors and clinical characteristics in the development of symptoms.

**Objectives:**

Investigation of the prevalence of ADHD symptoms in bipolar patients compared to the control group.Investigating differences in age of onset, clinical presentation, and course of affective illness between bipolar disorder patients with childhood ADHD symptoms compared to those without childhood ADHD symptoms.

**Methods:**

The study included 20 patients with bipolar disorder, hospitalized in the Psychiatric Hospital accompanied by their parents/relatives and 30 healthy controls (matched age, sex, socio-economic status) recruited through avalanche sampling in the Directorate of QSU “Mother Tereza”. The Abbreviated International Neuropsychiatric Interview (MINI) was used to identify cases with bipolar disorder or possible psychiatric pathology. The Diagnostic Interview for ADHD Adults (DIVA 2.0) was used to explore the presence of symptoms of attention deficit hyperactivity disorder (ADHD) in childhood and at the current age.Descriptive analysis in SPSS was used for data analysis.

**Results:**

It resulted that 80% of bipolar patients had ADHD symptoms in childhood compared to 16.67% in the control group. The age of onset of bipolar disorder was 17.31 years earlier in the group of cases with history of ADHD in childhood compared to the age of 21.25 years in cases without ADHD in childhood. In cases with history of ADHD, 43.75% had longer duration of manic episodes/ hypomanic compared to 25% in the group of cases without childhood ADHD. The number of suicide attempts 1 or >1 was more frequent in patients with a history of ADHD (25% and 12.5 %) compared to cases without a history of ADHD (25 %). In psychotic symptoms during mania, it was found that in patients with a history of ADHD in childhood, the prevalence was higher (81.82%) compared to (50%) in those without a history of ADHD in childhood. The prevalence of adult ADHD in cases was 35% compared to controls 6.66%. Apparently, the number of cases with comorbid ADHD with impaired social/family functioning was higher compared with the group of controls with comorbid ADHD.

**Conclusions:**

The associations of each clinical component of bipolar disorder with the presence or absence of ADHD in childhood were not statistically significant. However, it is worth noting that in complexity, the number of patients with more severe features of bipolar disorder is higher in cases with ADHD in childhood compared to cases without ADHD in childhood in our sample.

**Disclosure of Interest:**

None Declared

